# Electric-field assisted 3D-fibrous bioceramic-based scaffolds for bone tissue regeneration: Fabrication, characterization, and *in vitro* cellular activities

**DOI:** 10.1038/s41598-017-03461-x

**Published:** 2017-06-09

**Authors:** Minseong Kim, Hui-suk Yun, Geun Hyung Kim

**Affiliations:** 1 Department of Biomechatronic Engineering, College of Biotechnology and Bioengineering, Sungkyunkwan University (SKKU), Suwon, South Korea; 2 Powder and Ceramics Division, Korea Institute of Materials Science (KIMS), Changwon, South Korea

## Abstract

Nano/microfibrous structure can induce high cellular activities because of the topological similarity of the extracellular matrix, and thus, are widely used in various tissue regenerative materials. However, the fabrication of a bioceramic (high weight percent)-based 3D microfibrous structure is extremely difficult because of the low process-ability of bioceramics. In addition, three-dimensional (3D) microfibrous structure can induce more realistic cellular behavior when compared to that of 2D fibrous structure. Hence, the requirement of a 3D fibrous ceramic-based structure is an important issue in bioceramic scaffolds. In this study, a bioceramic (α-TCP)-based scaffold in which the weight fraction of the ceramic exceeded 70% was fabricated using an electrohydrodynamic printing (EHDP) process. The fabricated ceramic structure consisted of layer-by-layered struts entangled with polycaprolactone microfibers and the bioceramic phase. Various processing conditions (such as applied electric field, flow rate, nozzle size, and weight fraction of the bioceramic) were manipulated to obtain an optimal processing window. A 3D printed porous structure was used as a control, which had pore geometry similar to that of a structure fabricated using the EHDP process. Various physical and cellular activities using preosteoblasts (MC3T3-E1) helped confirm that the newly designed bioceramic scaffold demonstrated significantly high metabolic activity and mineralization.

## Introduction

In general, as an excellent convincing material, autologous bone substitute has been successfully applied in bone regeneration because of its low immune response and excellent osteoconducitve/osteoinductive properties^1^. However, the substitute unfortunately possesses certain clinical shortcomings including insufficient supply, long-term-pain at the donor sites, and secondary diseases (i.e., iatrogenic fracture and infection.)

Hence, new synthetic bone graft materials are required to successfully regenerate bone. In order to fulfill this demand, several biomedical scaffolds that incorporate a wide range of biomaterials were developed from bioceramics and synthetic/natural polymers that embed various growth factors, proteins, and cells in their hybrid composites. Among these biomaterials, bioceramics based on calcium phosphate are considered as significantly efficient materials due to the similarity in the structures of crystallites and chemical structure of bone apatite. Thus, they induce rapid bone formation due to the released ions, which are a prerequisite for efficient bone mineralization. Therefore, several studies confirmed that the pore-structure controlled calcium-phosphate-scaffolds promoted rapid osteogenesis and efficient osteointegration^2, 3^.

Although calcium-phosphate ceramic is considered as an outstanding bioactive material, the low fragility, flexibility, and low process- or shaping-ability of the ceramic pose obstacles to the fabrication of specifically designed porous scaffolds desirable for bone regeneration. It is widely-known that the pore architecture of biomedical scaffolds to regenerate bones is a significant factor because the pore structure (pore size, porosity, pore-interconnectivity, and tortuosity etc.) can significantly affect osteoconductivity and even angiogenesis. In order to overcome the shortcomings of calcium-phosphate ceramics, various studies focused on developing ceramic-based composites or hybrid structures using several synthetic (poly(lactic acid), poly(lactic-co-glycolic acid), and poly(ε-caprolactone) (PCL) etc.) and natural polymers (collagen, alginate, chitosan, and silk fibroin etc.)^3–6^. Among the synthetic polymers, the composites using the PCL were widely applied in bone regeneration because the PCL exhibited relatively slow biodegradation, reasonable elastic properties, and low inflammatory response^7^. Most studies using the PCL/ceramic composites focused on improving mechanical properties of the composites. However, the enhancement of *in vitro* cellular activities (initial cell adhesion, growth, and differentiation) using the composite continues to generate conflict. A few studies indicated that the composites enhanced osteoblastic differentiation, while other studies suggested that the composite did not improve biological responses. According to Barrere *et al*., calcium-phosphate ceramics like HA and TCP are known as highly bioactive materials to regenerate bone tissue, but the detail mechanism has not been completely revealed^8^. Since there are several types of bioceramics with difficulty of bonding with the bone tissue^9^ and the composition of the ceramic and biopolymer can affect the biological outcome of the composite^10, 11^, cellular activities including differentiation can be varied in each biocomposite. In recent, Duan *et al*. used two types of ceramic-based composites, Ca-P/poly(hydroxybutyrate–co-hydroxyvalerate) (PHBV) and carbonated hydroxyapatite (CHAp)/poly(l-lactic acid) (PLLA) composite^11^. The mechanical properties have shown increased in both of the biocomposites, compared to the pure polymer structures, while the cellular activities and differentiation differed in the two composite. These results show that the ceramic/polymer composites obviously can increase the mechanical properties, whereas they are not always enhancing the biological activities.

Here, a new calcium-phosphate-based composite with outstanding cellular activities was proposed. Generally, calcium phosphate ceramics (hydroxyapatite and tri-calcium phosphate (TCP)) are widely applied due to their outstanding biocompatibility, gradual degradation, and osteoconductivity^12^. The TCP has two main clear crystal phases, namely α-TCP and β-TCP phases. Although the two TCPs possess similar chemical composition, the solubility of α-TCP is considerably more rapid when compared to that of the β-TCP. Several studies^13, 14^ indicated that the α-TCP displayed histologically higher initial solubility and rapid *in vivo* bone formation when compared to those of the β-TCP. In order to obtain a composite material in this study, the material (α-TCP and PCL) and the electrohydrodynamic printing (EHDP) process supplemented with an ethanol were employed and used to fabricate microsized PCL fibers.

In recent, various electrostatic direct writing methods using an initial jet have been investigated. Sun *et al*. developed a near-field electrospinning process to deposit a nanosized poly(ethylene oxide)(PEO) fiber (20–500 nm) by applying a voltage of 600 V and the distance (50 μm) between an electrode and ground at 500 μm^15^. Another technique is a melt electrospinning writing process in which minimum fiber diameter of 5 μm can be deposited^16^. Both methods are outstanding techniques to attain position-controllable deposition using a single jet. However, the methods are applicable only to pure polymers (PEO and PCL), and these techniques may be difficult to apply biocomposites containing high weight fraction of the bioceramic. Furthermore, it may take a lot of time to fabricate a macroscale 3D porous structure.

A previous our study designed the EHDP process to obtain highly bioactive multi-layered PCL or cellulose fibrous bundles^17, 18^. These in turn provided excellent efficiency for cells (MC3T3-E1) such that they could easily attach and proliferate within the scaffold to guarantee sufficient nutrient supply and remove metabolic wastes generated by the entangled microfibrous structure^17^. However, the fabricated 3D fibrous structure only consisted of pure synthetic polymers, and did not fully provide osteoconductivity and realistic bone mimetic structure^11–14^.

In this study, various processing conditions of the EHDP process (such as processing geometry, applied electric field, flow rate, and nozzle size) were manipulated to fabricate a bone-mimetic ceramic-based microfibrous scaffold in which the weight fraction of the bioceramic exceeded 70%. The fibrous morphological structure, protein absorption ability, cement reaction, and mechanical properties of the composite scaffold fabricated using the EHDP process were compared with those of the scaffold fabricated using a conventional 3D printing process. In addition, preosteoblasts (MC3T3-E1) cellular activities (cell attachment, proliferation, and osteogenic gene expressions indicating mineralization) with respect to the composite were studied.

## Results and Discussion

### Selection of optimal processing conditions for fabricating α-TCP/PCL fibrous biocomposite

**Figure 1:**
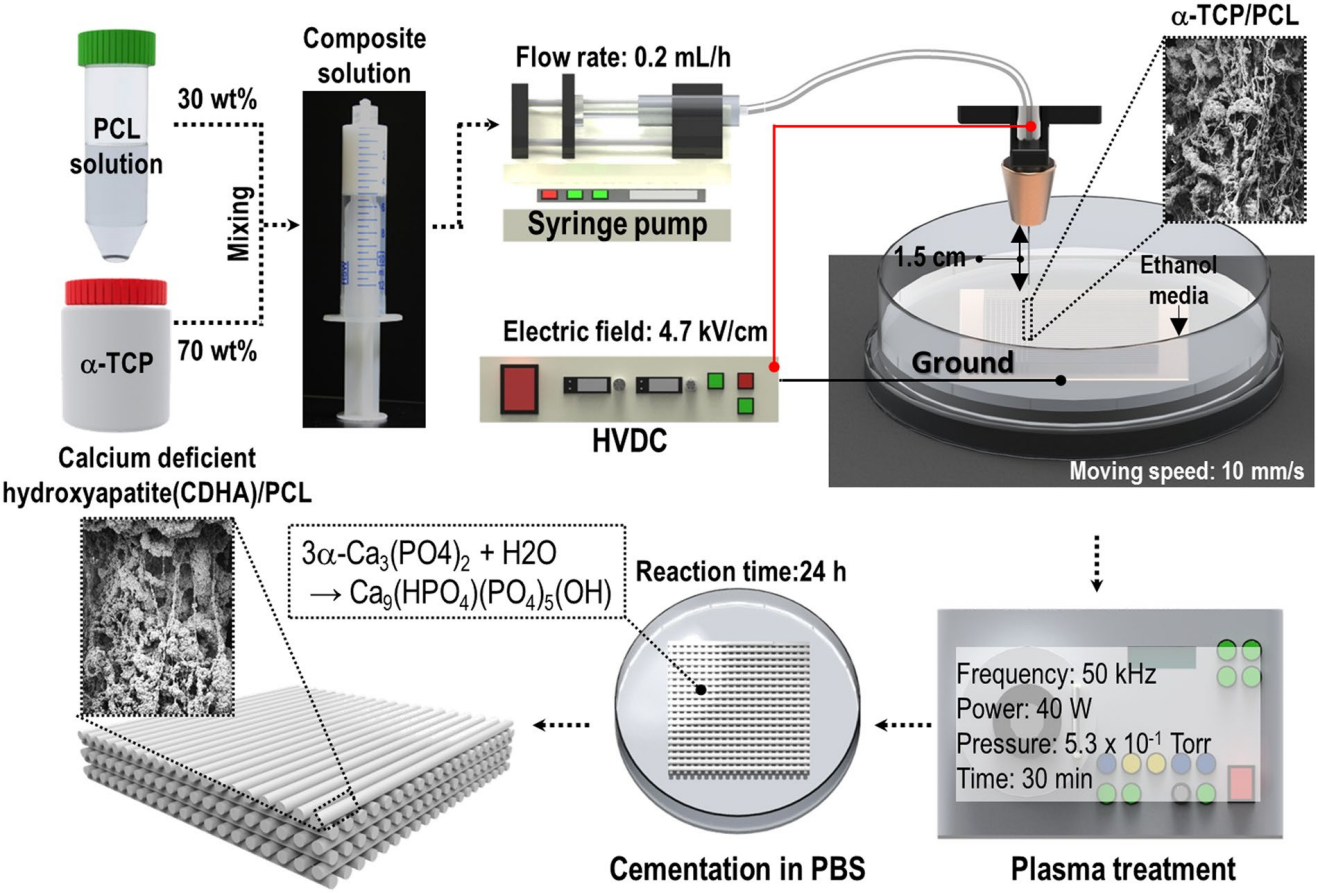
Schematic of an electrohydrodynamic printing process (EHDP) to obtain a fibrous ceramic-based scaffold.

The schematic description of the EHDP is presented in Fig. 1. In a manner different from general electrospinning, the method involved the use of a charged initial jet. Additionally, the charged single jet was changed into microsized fibrous bundles by replacing the solvent of the polymeric solution with the ethanol (EtOH) in the target bath, which possessed relatively low surface tension^17^. A previous study demonstrated fabricating efficiency to obtain fibrous bundles for the pure PCL and cellulose solution by controlling various processing parameters (such as applied electric field, feeding rate, and weight fraction of the polymer). Among these parameters, the applied electric field, nozzle size, flow rate of feeding solution, and height of the initial jet could significantly affect the development of microfibrous and multi-layered mesh structures.

**Figure 2:**
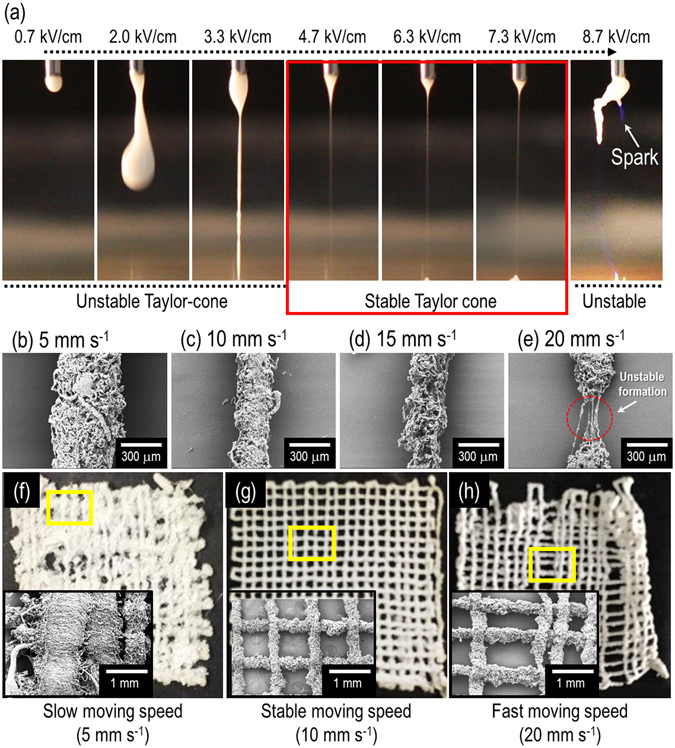
(**a**) Optical images of an initial jet exited from the nozzle tip for various electric fields (0.7~8.7 kV/cm) with respect to a height of 1.5 cm. The red region indicates the stable Taylor-cone and initial jet behavior. SEM images showing single struts fabricated at (**b**) 5 mm s^−1^, (**c**) 10 mm s^−1^, (**d**) 15 mm s^−1^, and (**e**) 20 mm s^−1^ of moving speed. Optical and SEM images of the mesh structure fabricated with the moving speeds, (**f**) 5 mm s^−1^, (**g**) 10 mm s^−1^, and (**h**) 20 mm s^−1^.

In order to observe the effect of an electric field on the fabrication, several electric field conditions (approximately in the range of 0.7–8.7 kV/cm) were used. In this study, the weight fraction of α-TCP was fixed as 70% because the volume percent of inorganic components in the real bone could exceed 55 vol%^19^. As shown in Fig. 2, optical images show the initial jet from the nozzle tip under various electric fields, a fixed height (15 mm) between the nozzle tip and surface of EtOH, and a constant flow rate (0.2 mL/h). As shown in the images, a stable Taylor-cone was obtained in the range of the electric field conditions (4.7~7.3 kV/cm), and the lower and upper ranges of the electric fields could result in the unstable development of Taylor cone or electric sparks between the nozzle tip and EtOH. Hence, the electric field condition was fixed as 4.7 kV/cm. Also, moving speed of the nozzle affected the scaffold morphology^20, 21^. Therefore, as shown in Fig. 2(b–e), the struts were fabricated with various moving speeds: 5, 10, 15, and 20 mm s^−1^. When the moving speed was 5 mm s^−1^, the accumulated fibrous struts was observed due to the slow moving speed of the nozzle (Fig. 2(b)). When the moving speed was relatively high (20 mm s^−1^), unstable formation of the strut was observed (Fig. 2(e)). However, for the moving speeds (10 and 15 mm s^−1^), stable formation of the struts was obtained (Fig. 2(c,d)). In addition, as seen in Fig. 2(f–h), the mesh structures using the moving speeds (5, 10, and 20 mm s^−1^) was fabricated. As expected, the mesh structure fabricated with the moving speed of 10 mm s^−1^ showed a stable mesh structure. Based on the result, we fixed the nozzle moving speed at 10 mm s^−1^.

**Figure 3:**
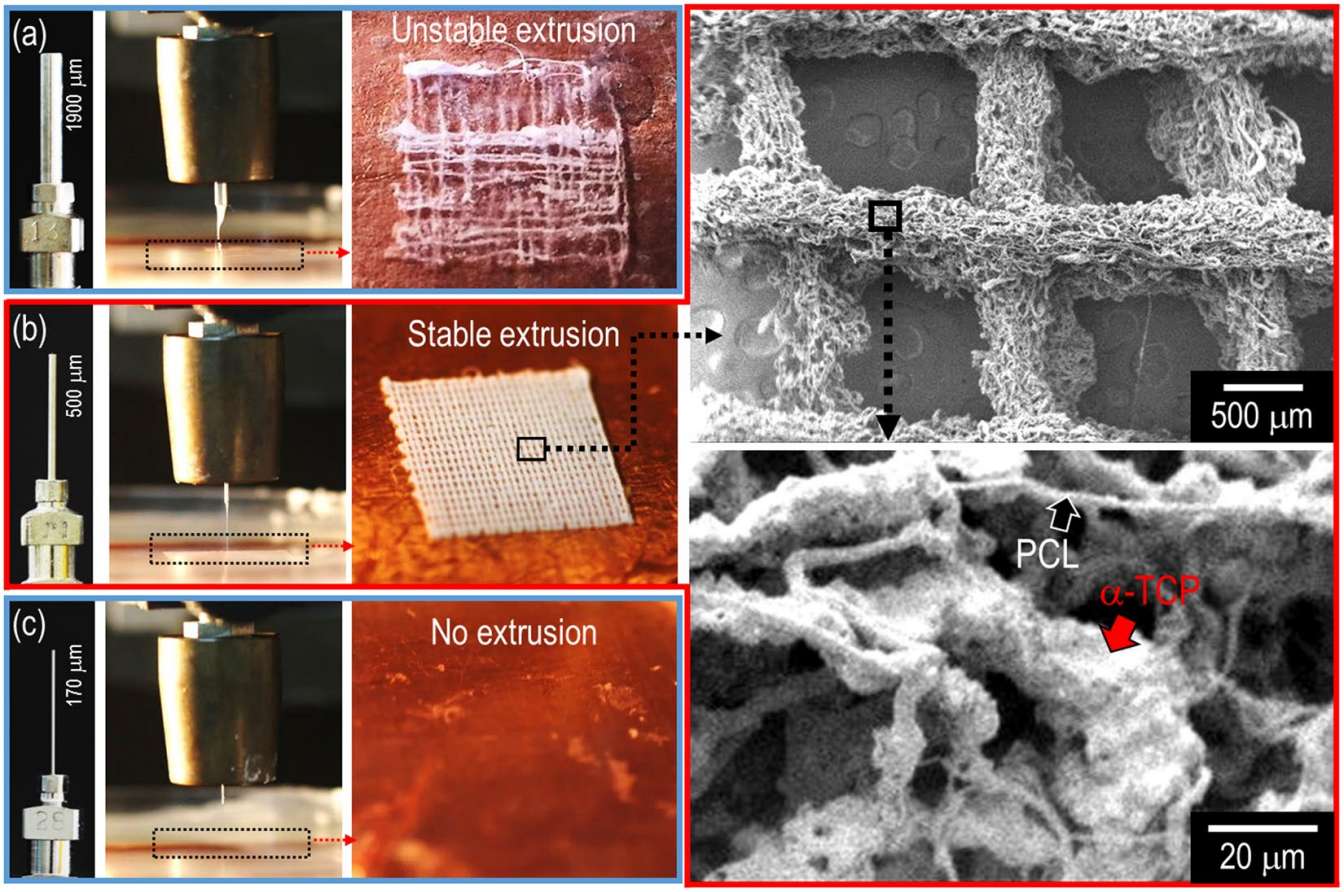
Effect of nozzle diameters ((**a**) 1.9 mm, (**b**) 0.5 mm, and (**c**) 0.17 mm) on the flow stability and the formation of layer-by-layered mesh structure. In the SEM image, stably deposited struts consisting of entangled microfibers of the mixture (ceramic/PCL) are described. The PCL corresponds to a fibrous form, and the ceramic corresponds to an agglomerated shape that is attached on the PCL fibers.

In order to observe the effect of nozzle diameter on the α-TCP/PCL solution, three different nozzle diameters were used, namely 13 G: 1.9 mm, 21 G: 0.5 mm, and 28 G: 170 μm under a fixed electric field of 4.7 kV/cm, height (15 mm), and flow rate of 0.2 mL/h. Figure 3(a–c) illustrate the optical images in the Taylor-cone and printed structure in the EtOH bath. Although a stable electrospinning process for pure polymeric solution or a polymer mixture with a low concentration of dispersed phase could be acquired given larger nozzle diameters, the increased size of the nozzle in the EHDP process could cause unstable deposition on the EtOH bath due to non-homogeneous or agglomerated flow of the ceramic mixture involving a high weight fraction of ceramic powders. Additionally, with respect to a considerably smaller nozzle size, the ceramic solution did not flow due to the aggregation and blockage of the ceramic particles in the nozzle. From the result, it was observed that an appropriate nozzle size was required, and the nozzle size was fixed as 21 G for future studies. Furthermore, as shown in the SEM images (magnified via the optical image) of the mesh structure fabricated with the nozzle size of 500 μm, the combinational structure consisting of microsized PCL fibers and dispersed ceramic powders was easily obtained with a macroscale mesh structure.

**Figure 4:**
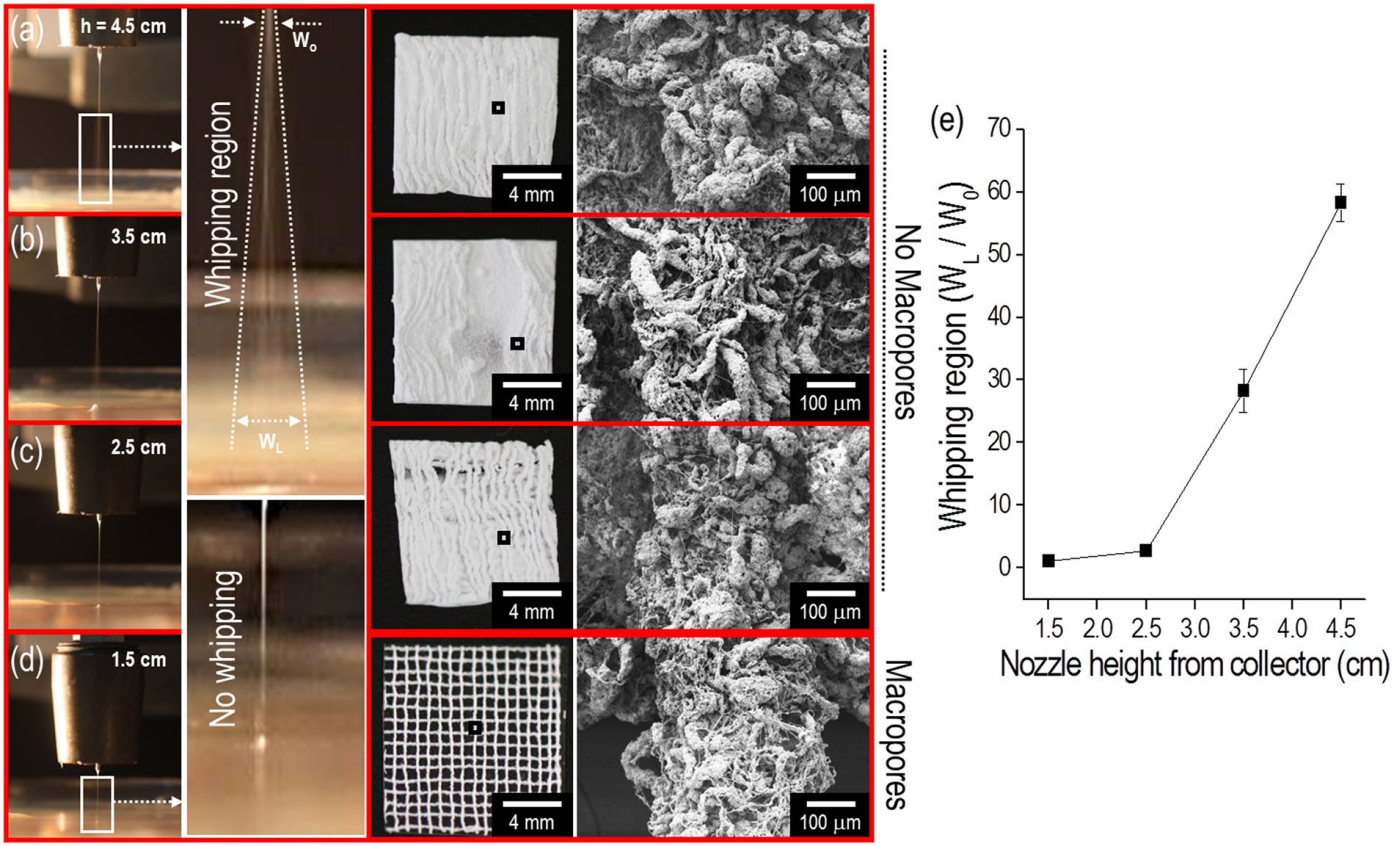
Effect of heights ((**a**) 4.5 cm, (**b**) 3.5 cm, (**c**) 2.5 cm, and (**d**) 1.5 cm) between a nozzle tip and surface of ethanol in the EHDP process under a constant electric field (4.7 kV/cm) on the formation of a layer-by-layer mesh structure. Magnified optical images show that the whipping and non-whipping behavior were obtained for different heights. (**e**) Relative whipping region (W_L_/W_o_) for various heights.

It is necessary to select an appropriate printing process to achieve a successful multi-layered macroscale mesh structure. Specifically, the height between the nozzle tip and the surface of EtOH in the EHDP method could be a highly important processing parameter since the mesh structure with controllable macropores cannot be achieved if whipping of the initial single jet occurred. Hence, the formation of the mesh structure was observed by changing the height (approximately in the range of 1.5–4.5 cm) of the process under the same electric field of 4.7 kV/cm. Figure 4(a–d) shows the initial single jet and whipping phenomenon for various heights. The whipping phenomenon was calculated with “W_L_/W_o_”, where W_L_ denotes the 2D whipping width captured in the optical image, and W_o_ denotes the diameter of initial single jet. As shown in the Fig. 4(e), the W_L_/W_o_ was gradually increased with increases in the height. Based on the result, we can confirm that the initial single jet was broken into a whipping motion at heights exceeding 1.5 cm such that the mesh structure was not attained. From the results, an appropriate height (1.5 cm) of the process could be selected to achieve the successful mesh structure.

**Figure 5:**
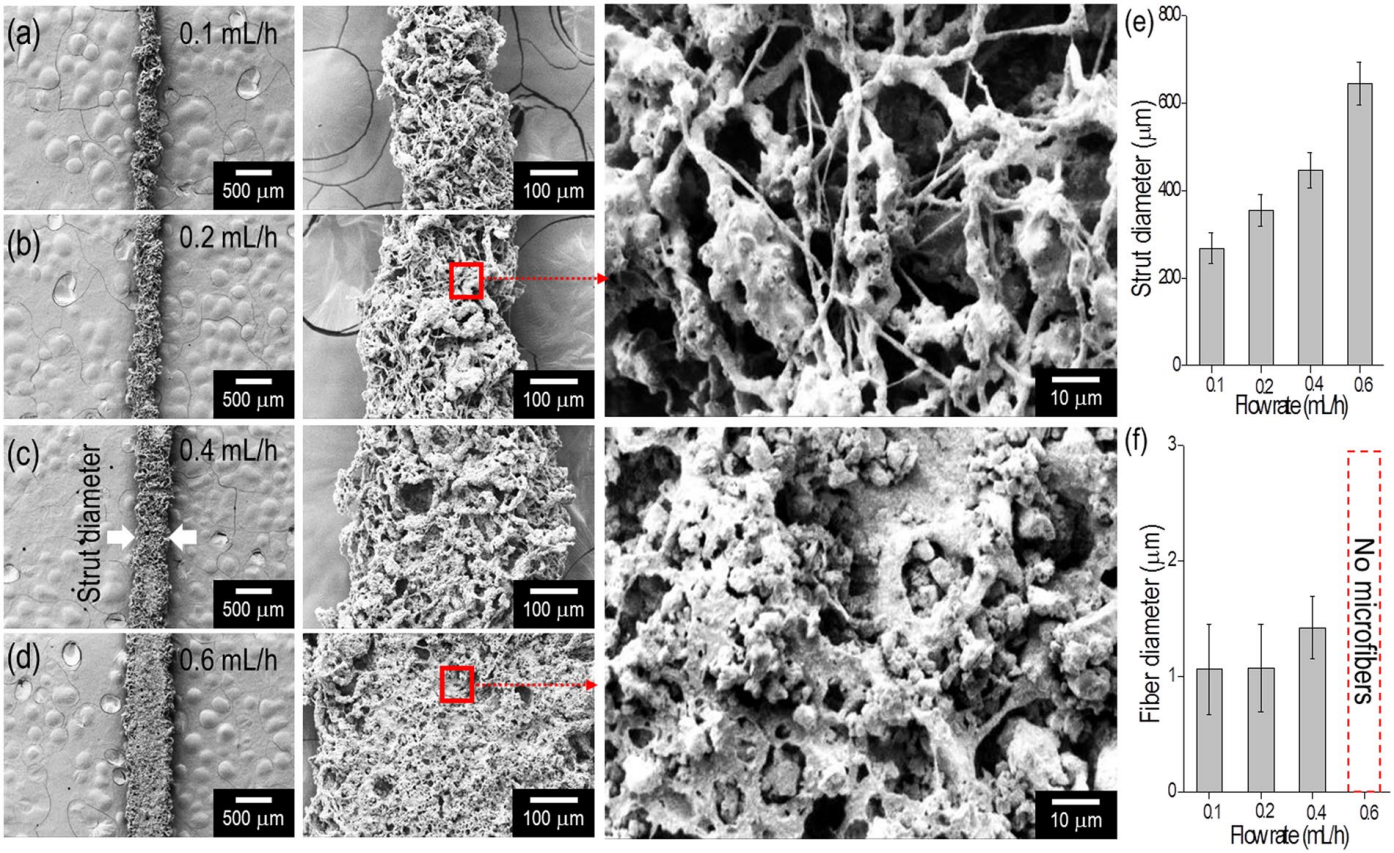
The effect of various flow rates ((**a**) 0.1 mL/h, (**b**) 0.2 mL/h, (**c**) 0.4 mL/h, and (**d**) 0.6 mL/h) of the ceramic mixture on the formation of fibrous structure of the printed strut. (**e**) Diameter of fabricated struts and (**f**) diameter of the constituting microfibers for various flow rates.

Another important processing parameter of the EHDP method corresponds to the flow rate of the feeding solution. Previous studies indicated that the diameter of fabricated macrosized strut consisting of PCL microfibers increased as the flow rate of the pure PCL solution increased^17^. In order to observe the effect of the feeding rate on the diameter of ceramic/PCL-based struts, the strut diameter consisting of microfibers was measured for several feeding rates (approximately in the range of 0.1–0.6 mL/h) in Fig. 5(a–d). As expected, the diameter of macro-struts increased with increases in the feeding rate (Fig. 5(e)), while the diameter of the PCL microfibers was similar (Fig. 5(f)) due to similar charges in the microfibers. Additionally, as shown in the magnified SEM images, the fibrous structure that was developed by the PCL component under the flow rate of 0.2 mL/h was well formed. However when the feeding rate exceeded 0.4 mL/h, the PCL fibrous structure was not fully developed due to the high mass flow rate of the ceramic solution. The feeding rate of the solution was selected as a 0.2 mL/h based on the results.

**Figure 6:**
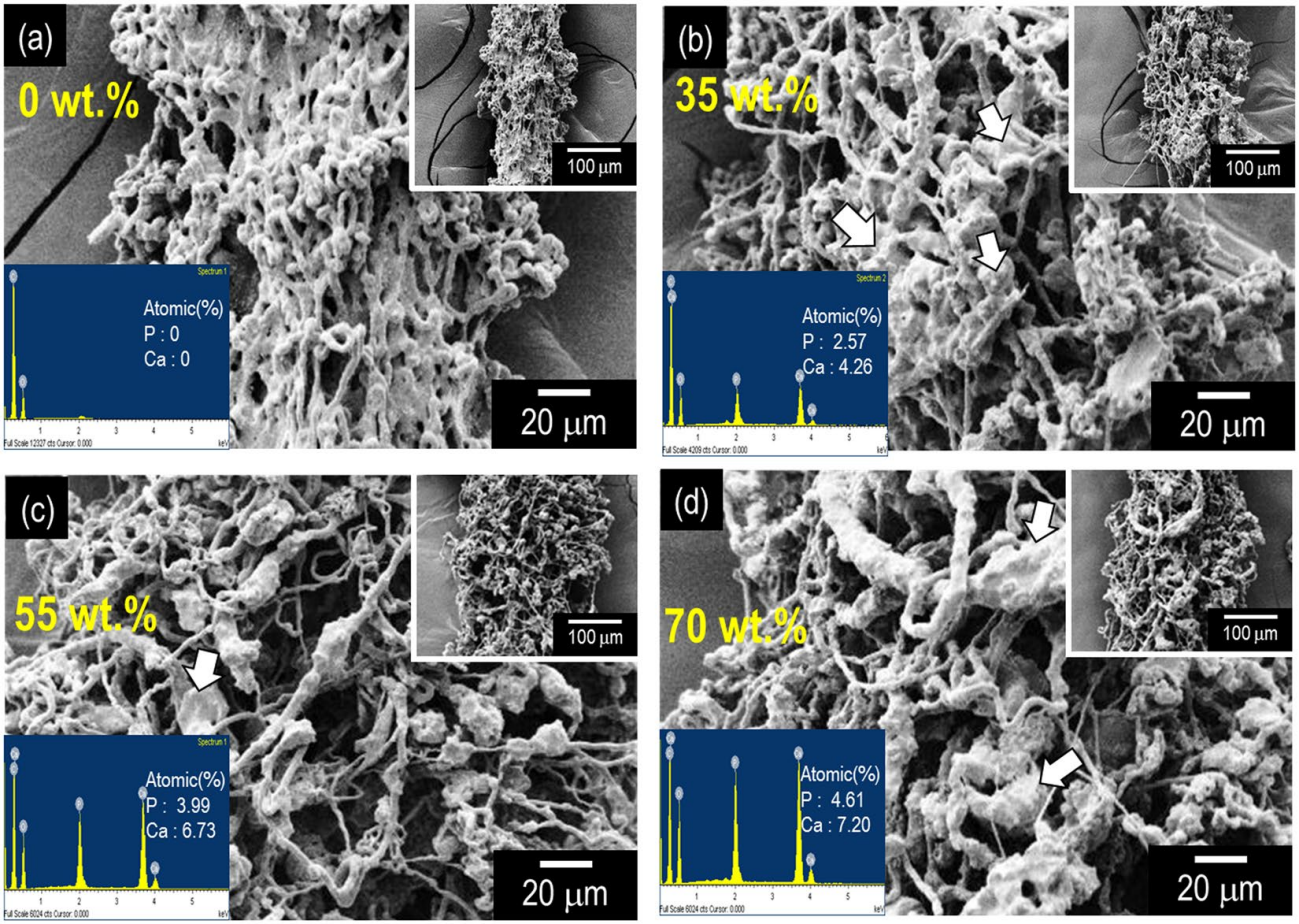
SEM images describing the ability of strut formation and EDS results for various weight fractions of the ceramic ((**a**) 0 wt.%, (**b**) 35 wt.%, (**c**) 55 wt.%, and (**d**) 70 wt.%) under selected EHDP process conditions.

Four different weight fractions, namely 0 wt%, 35 wt%, 55 wt%, and 70 wt% of α-TCP, were used to observe the effect of the weight fraction of α-TCP on the morphological structure of the fabricated struts. Figure 6(a–d) show the SEM and EDS results of fabricated struts obtained by using various weight fractions. As shown in the SEM images, the PCL microfibers were entangled for the pure PCL. Additionally, the region of α-TCP composition was enlarged as the weight fraction of the α-TCP increased (EDS result). However, at weight fractions exceeding 80 wt% of α-TCP, the process was not achieved due to the exceedingly high shear viscosity of the mixture.

**Figure 7:**
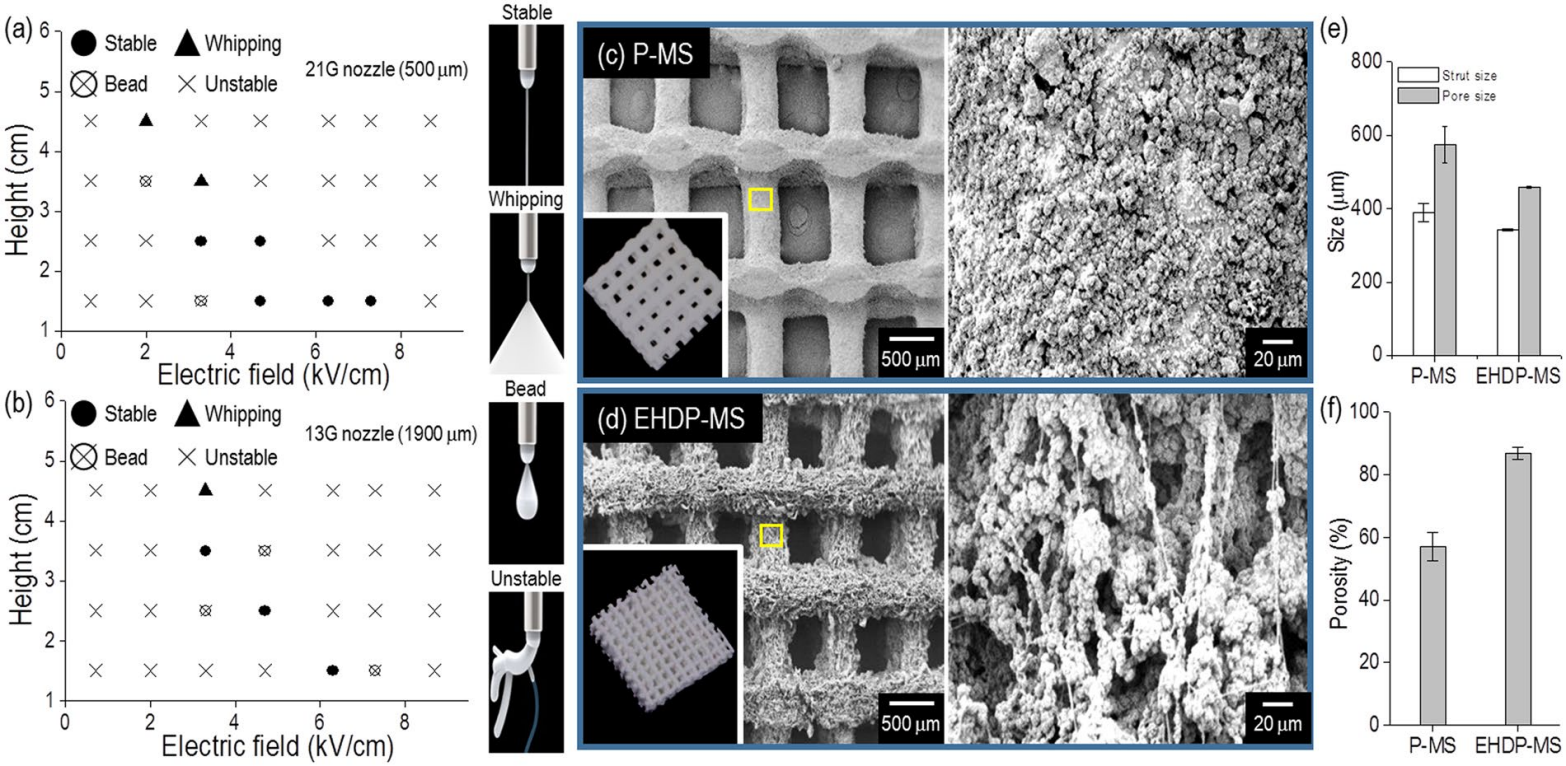
Processing window showing stable processing region for two nozzle diameters (**a**): 500 μm and (**b**): 1900 μm. SEM images of fabricated (**c**) P-MS and (**d**) EHDP-MS structures with slightly different strut diameters and pore sizes. (**e**) Average size of strut and pore and (**f**) porosity of the fabricated mesh structures.

Figure 7(a,b) demonstrate the processing window for 70 wt% α-TCP to obtain the stable fibrous struts and mesh structure, which are depending on several process factors: applied electric field (1~9 kV/cm), height (1.5~4.5 cm) between the nozzle tip and ethanol surface, and nozzle diameter (21 G and 18 G). As shown in the results, the stable processing range can be varied with each processing condition. Based on the previous processing results, the optimal processing parameters (applied voltage: 4.7 kV/cm, flow rate: 0.2 mL/h, a height between a nozzle and surface of EtOH: 1.5 cm, wt% of α-TCP: 70) could be selected to successfully achieve a controllable mesh structure consisting of ceramic-based microfibers.

### Fabrication of ceramic-based 3D-printed and fibrous scaffolds

In biomedical scaffolds, the pore and morphological structure including pore size, porosity, pore-interconnectivity, and tortuosity are the most important design factors because the geometrical size can directly affect the supply of nutrients and removal of metabolic wastes and eventually result in various cellular responses^22^. A typical pore geometry (with an average pore size exceeding 100 μm) for bone tissue regeneration was recommended by previous studies^22, 23^. Additionally, pore interconnectivity and tortuosity could influence cell infiltration and proliferation. Several studies^24, 25^ indicated that PEO (polyethylene oxide)-leaching or laser-induced macropores were accommodated to efficiently induce the cell-infiltration on 2D or 3D electrospun fibrous mats. The designed macro-pores in the fibrous structure exhibited the outstanding infiltration or migration of injected cells (MSCs and osteoblast-like-cells).

Furthermore, surface topological structure on the biomedical scaffolds could affect initial cell adhesion and proliferation. According to Zhu *et al*., SaOS-2 osteoblast-like cells were cultured on both porous titanium surfaces and polished/anodized titanium surfaces, and the structure with porous surface roughness exhibited outstanding cell adhesion and proliferation^26^.

Figure 7(c,d) shows optical and SEM images of a generally printed 3D mesh structure (P-MS) and an EHDP-mesh structure (EHDP-MS) that was fabricated using the selected processing conditions. In the structures, the diameter of the strut was 389.2 ± 25.2 µm (P-MS) and 341.9 ± 18.9 µm (EHDP-MS), and the pore size was 574.9 ± 49.7 µm (EHDP-MS) and 459.1 ± 33.8 µm (EHDP-MS), respectively (Fig. 7(e)). The size of the struts and pore of the two mesh structures were not completely similar, but if we consider the different fabrication methods (pneumatic pressured (P-MS) and syringe pumping (EHDP-MS) system), the size different can be negligible. However, the porosity of the EHD-MS and P-MS was completely different (Fig. 7(f)). Additionally, a comparison of the magnified SEM images of P-MS and EHDP-MS indicated that the surface of the struts was completely different. The surface of EHDP-MS showed a significantly porous structure, which was mixed with fibers and aggregated ceramic pastes, while, the surface of P-MS was relatively smooth. Based on the analysis, the EHDP-MS possessed two different scale porous structures, namely (1) macropores between the struts to induce nutrient transport or efficient cell infiltration/migration, and (2) microporous on the struts to enhance high initial cell adhesion/proliferation. Therefore, it was estimated that the EHDP-MS provided highly proficient cellular activities.

### Characterization of the α-TCP/PCL fibrous biocomposite

The ceramic-based mesh structure (P-MS) was used as a control to observe the physical and biological characterizations of the fibrous biocomposite (EHDP-MS).

**Figure 8:**
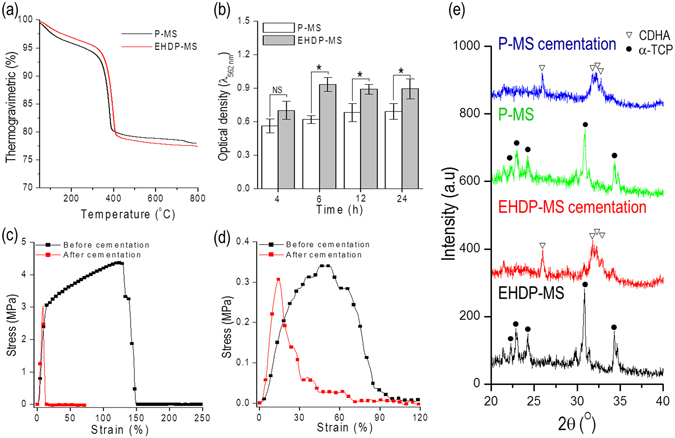
(**a**) Thermogravimetric analysis of P-MS and EHDP-MS, (**b**) protein absorption of P-MS and EHDP-MS, stress-strain curves of (**c**) P-MS and (**d**) EHDP-MS before and after cement reaction, and (**d**) XRD results of P-MS and EHDP-MS before and after cement reaction.

Figure 8(a) shows the TGA result specifying the degradation temperature of PCL that was approximately in the range of 370–390 °C and the remnant ceramic, namely α-TCP. As shown in the results, the remnant wt % (α-TCP) of P-MS and EHDP-MS approximately corresponded to 72 and 77, respectively. Although each scaffold was fabricated using different fabricating process such as pneumatic pressured (P-MS) and syringe pumping (EHD-MS) system, it could be assumed that the embedded weight percent of α-TCP in both scaffolds was similar.

The capability of protein absorption of a biomedical scaffold is considered as one of important indicators for various cellular activities including cell-adhesion and migration due to the cell-binding proteins (e.g., vitronetine, fibronectin, and fibrinogen)^27^. Generally, the protein absorption in the scaffold is directly related to properties such as surface topography, ionic interaction, and hydrophobicity^28^. Figure 8(b) shows the capability of protein absorption for the P-MS and EHDP-MS scaffold. As shown in the results, the capability of the protein absorption of the EHDP-MS was meaningfully higher when compared with that of the P-MS. This phenomenon occurred because the microfibrous structure of EHDP-MS could provide a much larger contact surface-area for the proteins when compared with that of the P-MS. Based on this simple result, it was estimated that with respect to the EHDP-MS, initial cell-adhesion and migration were effectively achieved.

Mechanical stiffness of biomedical scaffolds was a significant parameter because it affected cellular activities (e.g., cell morphology and differentiation) and also enabled the cell to endure various external mechanical stresses^29^. Figure 8(c,d) shows the tensile stress-strain curves of P-MS and EHDP-MS under a stretching speed corresponding to 0.5 mms^−1^ in a dry state. Additionally, as shown in the data, the tensile property was measured before and after the cementation process of the scaffolds. The P-MS displayed a Young’s modulus of 42.45 ± 0.76 MPa before and 55.3 ± 7 MPa after the cementation. In contrast, with respect to the EHDP-MS, the modulus was 1.65 ± 0.34 MPa before and 3.36 ± 0.9 MPa after the cementation. Although the mechanical stiffness of the scaffolds improved after the cementation process, the measured mechanical stiffness of both scaffolds were still significantly low due to the high porosity of scaffolds relative to the actual trabecular bone (0.76 ± 0.4 GPa)^30^. Although the designed scaffold could perform the function of a temporary biological supporter (which should be degraded following implantation) of cell attachment, infiltration, and proliferation, it is clear to improve the low mechanical properties of the EHDP-MS.

Figure 8(e) shows the XRD data for P-MS and EHDP-MS before and after cementation in the PBS solution. As shown in the XRD data, both scaffolds exhibited the typical XRD pattern of α-TCP, which involved an orthorhombic crystal structure. Conversely, the hydrolysis occurred in the α-TCP structure after immersing the scaffolds in the PBS solution during 24 h. Eventually, the α-TCP was completely changed into calcium-deficient hydroxyapatite (CDHA), which had a crystal structure analogous to that of inorganic constituents in bones^31^. The CDHA peaks were observed clearly as shown in the XRD peaks of both scaffolds.

### *In vitro* cellular activities of biocomposite

**Figure 9:**
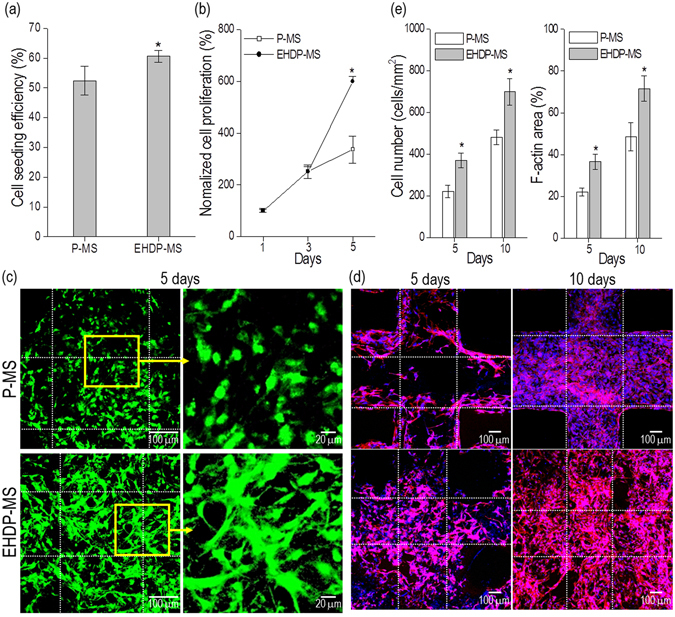
(**a**) Cell-seeding efficiently, (**b**) MTT result, (**c**) live/dead cells, and (**d**) DAPI/Phalloidin images of both structures (P-MS and EHDP-MS). (**e**) The number of nuclei and F-actin areas using DAPI/Phalloidin images.

Figure 9(a) shows the efficiency of cell-seeding for the two scaffolds, P-MS and EHDP-MS. As widely-known, efficiency is one of important factors in evaluating the scaffold because it is directly related with important cell losses. As shown in the results, the seeding efficiency of the EHDP-MS approximately corresponded to 62 ± 4% when compared to 52 ± 9% for the P-MS. The efficiency of P-MS was relatively high when compared to that of general synthetic polymers due to the hydrophilic property of the ceramic component. Additionally, the efficiency of EHDP-MS was significantly high due to the high surface to volume ratio of the struts that consisted of microfibers.

Figure 9(b) shows the growth of viable cells that was determined by the MTT assay of the P-MS and EHDP-MS. The number of viable cells of the EHDP-MS was significantly higher than that of P-MS at 1 d, 3 d, and 5 d. The cell-growth rate, which was calculated by a simple linear regression of cell-number relative to cell-culture day, of P-MS and EHDP-MS were approximately 5.0 × 10^4^ and 9.1 × 10^4^, respectively. Based on the result, it was confirmed that the cells in the EHDP-MS were more significantly proliferated than those in the P-MS, indicating that the EHDP-MS exhibited more meaningful metabolic activities. This phenomenon occurred because the cells in the EHDP-MS were efficiently attached and proliferated on the microfibrous porous struts because the fibrous unique bundle structure of EHDP-MS was activated as an outstanding cell-growing site to encourage effective micro-environmental cell-to-cell relations.

In Fig. 9(c,d), live (green)/dead (red) cell images and nuclei (blue)/cytoskeleton (red) of the cells for the mesh structures cultured for 5 d are shown. Figure 9(e) shows the number of nuclei per mm^2^ and the area of F-actin on both structures. As expected in the MTT result, the cultured cells in both structures were well alive, and the cells cultured on the EHDP-MS were more homogeneously proliferated and even fully stretched compared to the P-MS. This morphological phenomenon was because of the microfibrous structure on the macrosized struts of the EHDP-MS. The morphological unique structure can affect the cell mineralization because stretched cells can influence the mechano-transduction pathways, so that these phenomena can directly affect gene and protein expression of cell mineralization^32, 33^. Based on the result, we can estimate that the cells in the EHDP-MS may differentiate more efficiently than that in the P-MS.

**Figure 10:**
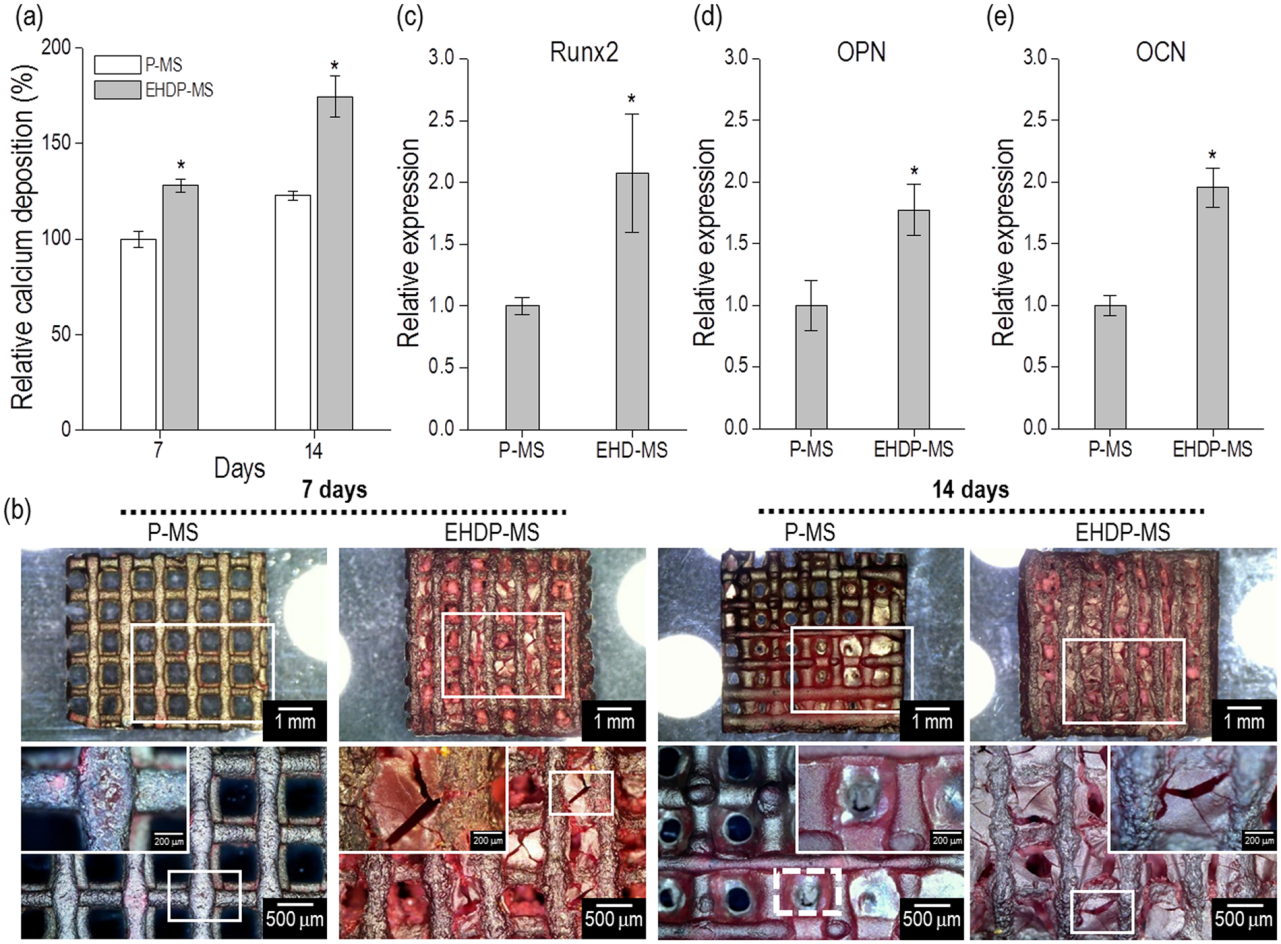
(**a**) Calcium deposition and (**b**) optical images of ARS staining of the P-MS and EHDP-MS following 7 d and 14 d of cell culture. Osteoblast differentiation marker genes, such as (**c**) Runx2, (**d**) OPN (osteopontin), and (**e**) OCN (osteocalcin) after cell-culture for 10 days.

In order to observe the osteogenic differentiation of the structures, the calcium deposition was measured and qualitatively obtained using alizarin red staining. As shown in Fig. 10(a), the calcium deposition of the scaffolds was determined after 7 d and 14 d. The measured optical density (OD) was subtracted from that of each scaffold, which was not cultured with the cells, and also normalized with respect to the total protein contents. As shown in the results, the value with respect to the EHDP-MS was meaningfully higher than with respect to the P-MS. Figure 10(b) shows the optical images that describe the stained scaffolds; the images demonstrate that a significant proportion of dense red-color was observed in the struts and even in the pores on the EHDP-MS, while comparatively less-intense areas of red-color were observed in P-MS. The result indicated that the EHDP-MS could be an extremely bioactive platform for bone tissue regeneration.

Furthermore, the differentiation of MC3T3-E1 was measured in both structures to assess the expression of osteoblast marker gene using reverse transcriptase-polymerase chain reaction (RT-PCR). Generally, the runt-related transcription factor 2 (RUNX2), osteopontin (OPN), and osteocalcin (OCN) have been known as typical osteogenic markers for the differentiation. As shown in the osteogenic gene expressions (Fig. 10(c–e)), it was confirmed that the EHDP-MS provided a significantly high biological cell-to-cell interaction, which affects ultimately cellular function^34^, for the osteoblastic cells when compared to that of the simply printed structure (P-MS). Consequently, the results once again evidently demonstrated that the presence of the 3D micro-fibrous structure could be decisive to induce a proper and meaningful 3D cell-culture environmental condition. It is expected that the meaningfully improved differentiation could be instigated from the combinational synergistic effect of high weight fraction of α-TCP with the morphological structure of EHDP-MS.

## Conclusion

In this study, a novel fibrous composite scaffold that consisted of PCL and high weight fraction of α-TCP was manufactured and assessed by using an electrohydrodynamic printing process supplemented with ethanol. In the study, a reasonable processing window for the various processing parameters, including electric field, processing height, flow rate, and even nozzle diameters, was selected. The selected processing conditions were used to safely fabricate a 3D mesh ceramic structure consisting of microfibrous PCL and ceramic component. The significant increase of various cellular activities, such as increased initial cell attachment, proliferation, and differentiation, of the structure confirmed that the newly designed fibrous ceramic scaffold possessed immense potential as a material for regenerating bone tissues.

## Experimental

### Fabrication of ceramic-scaffolds

In the study, α-TCP was kindly provided by Dr. H.-S Yun (Powder and Ceramics Division, Korea Institute of Materials Science, South Korea), and a dispersed material, PCL (density = 1.135 g/cm^3^; M_w_ = 80,000) was obtained from Sigma-Aldrich (St. Louis, MO, USA). 8 wt% PCL solution was used in a 20:80 solvent mixture of methylene chloride and dimethylformamide (Junsei Chemical Co., Tokyo, Japan), and it was mixed with various weight fractions of α-TCP.

A nozzle moving speed of 10 mm s^−1^ was controlled using a three-axis printing system (DTR3–2210 T-SG, DASA Robot, Seoul, South Korea). The supply rate of the mixture solution was controlled with a syringe pump (KDS 230; KD Scientific, Holliston, MA, USA). A power supply (SHV300RD-50K; Convertech, Seoul, South Korea) was used to provide the electric field. The detail fabrication schematic for the 3D ceramic-based scaffold is described in Fig. 1. As shown in the image, the EHDP used ethanol as a target medium, and the charged initial jet of the mixture (α-TCP/PCL) from the nozzle was directly immersed in the ethanol solution. Following the fabrication of the multi-layered structure, it was lyophilized using a freeze dryer (SFDSM06; Samwon, Busan, South Korea) at −75 °C for 1 d. After simple plasma treatment to induce hydrophilic property of PCL component, the fabricated mesh structures were immersed in preheated PBS for 24 h at 37 °C to perform the hydrolysis reaction, and the reacted structure was lyophilized again using the same condition.

### Characterization of the hybrid scaffolds

The surface morphology of the bioceramic scaffolds was characterized by scanning electron microscopy (SEM) (SNE-3000M, SEC Inc., Seoul, South Korea) and an optical microscope (Model BX FM-32; Olympus, Tokyo, Japan) connected to a digital camera. In addition, to observe the distribution of elemental calcium and phosphorus in the fibrous mesh structure, energy-dispersive spectroscopy (EDS) analyses were carried out

The pore size was defined as the distance between the fibrous bundles and was measured on the SEM images. Thermogravimetric analysis (TGA) was conducted under nitrogen using a TGA-2050 (TA-Instruments, New Castle, DE, USA). A typical sample mass of 10 mg was heated from 30 °C to 900 °C at a ramp rate of 20 °C min^−1^. Wide-angle X-ray diffraction (Siemens D500 WAXD, Munich, Germany) with CuKα radiation under beam conditions of 40 kV and 20 mA with collection of a spectrum at 2θ = 20~40° and a step size of 0.1° was performed to obtain crystal peaks of α-TCP and calcium-deficient hydroxyapatite (CDHA).

Protein absorption was measured using bicinchoninic acid (BCA) protein assay (Pierce Kit; Thermo Scientific, Waltham, MA, USA). Scaffolds (size: 6 × 6 × 1 mm^3^) were placed in 24 well plates containing α-minimum essential medium (α-MEM) and 10% fetal bovine serum (FBS) (Gemini Bio-Products, Calabasas, CA, USA) and incubated at 37 °C for 4 h, 6 h, 12 h, and 24 h. The specimens were washed with PBS and lysed with 0.1% Triton X-100. An aliquot of the lysate (25 μL) was added to 200 μL of BCA working reagent, and the mixture was incubated for 30 min at 37 °C. Absorbance at 562 nm was determined using a plate reader. Samples incubated in a serum-free medium were used as blanks. Protein adsorption values are presented as means ± SD (n = 5).

The scaffold was cut into small strips to assess the mechanical properties. A tensile test was performed using a universal tensile machine (Top-tech 2000; Chemilab, Seoul, South Korea). The stress–strain curves for the ceramic scaffolds were recorded at a stretching speed of 0.5 mm s^−1^. All values corresponded to means ± standard deviation (SD) (n = 5).

### *In vitro* cell culture

Scaffolds measuring 6 × 6 × 1 mm^3^ were prepared and sterilized using 70% ethanol for 1 h with a 2 h ultraviolet light application prior to overnight incubation in a culture medium. Mouse preosteoblast cells (MC3T3-E1; ATCC, Manassas, VA, USA) were cultured in the scaffolds and maintained in α-MEM (Life Sciences, Carlsbad, CA, USA) containing 10% FBS and 1% antibiotic–antimycotic (Cellgro, Herndon, VA, USA). The cells were seeded onto the scaffolds at a density of 1 × 10^5^ per specimen and incubated under an atmosphere of 5% CO_2_ at 37 °C with the medium exchanged every alternate day.

Cell-seeding efficiency was also measured. Briefly, the seeded cells were left in the ceramic-scaffolds for 12 h to provide sufficient time for the cells to adhere to the ceramic-scaffolds. After 12 h, the ceramic scaffolds were removed, and the cells remaining in the wells were counted. The efficiency for the ceramic scaffold was calculated by accounting for the initial number of cells that were seeded and the residual number of cells in the respective well after 12 h. Five specimens of each ceramic-scaffold were used. The seeding efficiency was calculated as follows: seeding efficiency (%) = (cells added to scaffold−cells in wells)/(cells added to scaffold) × 100.

The proliferation of viable cells was determined by the MTT assay (Cell Proliferation Kit I; Boehringer Mannheim, Mannheim, Germany). This assay was based on cleavage by mitochondrial dehydrogenases in viable cells of yellow tetrazolium salt, MTT, to produce purple formazan crystals. Cells on the surface were incubated with 0.5 mg mL^−1^ MTT for 4 h at 37 °C. Absorbance at 570 nm was measured using a microplate reader (EL800; BioTek Instruments, Winooski, VT, USA). Five samples were tested per incubation period, and each test was performed thrice.

Alizarin Red-S staining of MC3T3-E1 cells in 24-well plates determined the calcium mineralization of the samples. The cells were cultured in α-MEM containing 50 μg mL^−1^ vitamin C and 10 mM β-glycerophosphate. The cells were then washed thrice in PBS, fixed in 70% (v/v) cold ethanol (4 °C) for 1 h, and air dried. The ethanol-fixed specimens were stained with 40 mM Alizarin Red-S (pH 4.2) for 1 h and washed thrice with purified water. The samples were then destained with 10% cetylpyridium chloride in 10 mM sodium phosphate buffer (pH 7.0) for 15 min. An optical microscope was used to observe the staining.

Runt-related transcription factor 2 (Runx2), osteopontin (OPN), and osteocalcin (OCN) were measured using the real-time RT-PCR. After 10 days, total RNA from the cultured scaffolds was isolated using the TRIzol reagent (Sigma-Aldrich) according to the manufacture’s protocol. Complementary DNA (cDAN) was synthesizing from total RNA. The reverse transcription (RT) reaction was carried out using ReverTra Ace qPCR RT Master Mix (Toyobo, Japan). The cDNA was amplified with THUNDERBIRD SYBR qPCR Mix (Toyobo, Japan) using ABI Step One Plus, under the following conditions: denaturation at 95 °C for 1 min, followed by 40 cycles of 95 °C for 15 s, 60 °C for 60 s and 72 °C for 15 s, with a final extension at 72 °C for 5 min. Gene-specific primers were as follows: mouse runx2 (forward: 5′-ACATCCCCATCCATCCAT-3′, reverse: 5′-GGTGCTGGGTTCTGAATCTG-3′), mouse OPN (forward: 5′-GGAGGAAACCAGCCAAGG-3′, reverse: 5′-TGCCAGAATCAGTCACTTTCAC-3′), mouse OCN (forward: 5′-CCCTCCTGAAGGTCTCACAA-3′, reverse: 5′GCTGTCTCCCTCATGTGTTG-3′) and the housekeeping gene mouse GAPDH (forward: 5′-CCTTGAGATCAACACGTACCAG-3′, reverse: 5′-CGCCTGTACACTCCACCAC-3′).

### Fluorescence Images

After 5 d in the cell culture, the composite scaffolds were exposed to 0.15 mM calcein AM and 2 mM ethidium homodimer-1 for 45 min in an incubator to observe live and dead cells. The stained specimens were visualized under a microscope (TE2000-S; Nikon, Tokyo, Japan) equipped with an epifluorescence attachment and a SPOT RT digital camera (SPOT Imaging Solutions, Sterling Heights, MI). The stained images were captured, and the green and red colors indicated live and dead cells, respectively. ImageJ software (National Institutes of Health, Bethesda, MD) was used to count live cells.

Following 5 d and 10 d of cell culture, the scaffolds were subjected to diamidino-2-phenylindole (DAPI) fluorescence staining to detect cell nuclei. Phalloidin (Invitrogen, Carlsbad, CA) staining was also performed to visualize the actin cytoskeleton of proliferated cells. A laser-scanning microscope (LSM510; Carl Zeiss, Oberkochen, Germany) was used to obtain the images.

### Total protein content

The total protein content was measured by the BCA protein assay (Pierce kit; Thermo Scientific). The scaffolds were washed with PBS and lysed with 1 mL of Triton X-100 (0.1%). An aliquot of the lysate (25 μL) was added to 200 μL of BCA working reagent, and the mixture was incubated for 30 min at 37 °C. Protein concentration was determined from the absorbance at 562 nm that was measured using an enzyme-linked immunosorbent assay reader and converted to the total protein concentration using a standard curve.

### Statistical analyses

All data were presented as means ± standard deviation. Statistical analyses were performed using SPSS software (version 20.0; SPSS, Inc., Chicago, IL) and included single-factor analyses of variance (ANOVA). In all the analyses, *P* values < 0.05 were considered to indicate statistical significance. Furthermore, “NS” indicates no statistically significant difference.
